# The Automatic Assessment of Strength and Mobility in Older Adults: A Test-Retest Reliability Study

**DOI:** 10.3390/medicina55060270

**Published:** 2019-06-11

**Authors:** Daniel Collado-Mateo, Pedro Madeira, Francisco J. Dominguez-Muñoz, Santos Villafaina, Pablo Tomas-Carus, José A. Parraca

**Affiliations:** 1 Faculty of Sport Science, University of Extremadura, 10003 Cáceres, Spain; danicolladom@gmail.com (D.C.-M.); fjdominguez@unex.es (F.J.D.-M.); 2 Facultad de Educación, Universidad Autónoma de Chile, Talca 1670, Chile; 3 Departamento de Desporto e Saúde, Escola de Ciências e Tecnologia, Universidade de Évora, 7000-727 Évora, Portugal; pedromadeira94@hotmail.com (P.M.); ptc@uevora.pt (P.T.-C.); jparraca@uevora.pt (J.A.P.); 4 Comprehensive Health Research Centre (CHRC), University of Évora, 7000-727 Évora, Portugal

**Keywords:** intraclass correlation coefficient, standard error of measurement, older adults, reliability analysis, physical fitness tests

## Abstract

*Background:* Simple field tests such as the Timed Up and Go test (TUG) and 30 s Chair Stand test are commonly used to evaluate physical function in the elderly, providing crude outcome measures. Using an automatic chronometer, it is possible to obtain additional kinematic parameters that may lead to obtaining extra information and drawing further conclusions. However, there is a lack of studies that evaluate the test-retest reliability of these parameters, which may help to judge and interpret changes caused by an intervention or differences between populations. Thus, the aim of this study was to evaluate the test-retest reliability of the Timed Up and Go test (TUG) and 30 s Chair Stand test in healthy older adults. *Methods:* A total of 99 healthy older adults participated in this cross-sectional study. The TUG and the 30 s Chair Stand test were performed five times and twice, respectively, using an automatic chronometer. The sit-to-stand-to-sit cycle from the 30 s Chair Stand test was divided into two phases. *Results:* Overall, reliability for the 30 s Chair Stand test was good for almost each variable (intraclass correlation coefficient (ICC) >0.70). Furthermore, the use of an automatic chronometer improved the reliability for the TUG (ICC >0.86 for a manual chronometer and ICC >0.88 for an automatic chronometer). *Conclusions:* The TUG and the 30 s Chair Stand test are reliable in older adults. The use of an automatic chronometer in the TUG is strongly recommended as it increased the reliability of the test. This device enables researchers to obtain relevant and reliable data from the 30 s Chair Stand test, such as the duration of the sit-to-stand-to-sit cycles and phases.

## 1. Introduction

The age of the world’s population has increased and the proportion of older adults has rapidly grown in the last decades. The aging process leads to a reduction in physical function and muscular mass, as well as to a higher risk of disability, diseases, autonomy loss, and premature death [1]. In this regard, frailty is an age-associated health state characterized by vulnerability caused by aging (>65 years) and other processes [2].

Physical function in the elderly is crucial to autonomously perform activities of daily living [3,4]. The process of aging is particularly conditioned by healthy lifestyle habits, especially physical activity levels [5]. An objective evaluation of physical function is thus relevant to identify frail older adults who are at risk of losing their autonomy and independence [6]. In this regard, previous studies have identified muscle weakness as a factor related to a higher risk of falling [7,8,9,10].

Simple field tests such as the Timed Up and Go test (TUG) [9] and the 30 s Chair Stand test [10,11] are commonly used to evaluate physical function in the elderly. The score in the TUG is the time required to rise from a chair, walk as fast as possible towards a mark placed at 2.44 m (8 feet) from the chair, turn around, and sit again. In this test, the time recorded may be influenced by the ability of the rater [12]. Therefore, the use of automatic chronometers is recommended in order to remove the human variability of the rater and increase the reliability of the test. However, to our knowledge, there is no study aimed at evaluating the test-retest reliability of the TUG using automatic chronometers.

In the case of the 30 s Chair Stand test, the traditional test only provides a crude outcome measure (i.e., the number of times that a person is able to stand up from a chair and sit back), which may limit the clinical relevance of the test. Using the same automatic chronometer, it is possible to obtain additional kinematic parameters that may lead to obtaining extra information and drawing further conclusions [13]. Previous studies reported the association between kinematic performance of the 30 s Chair Stand test and frailty in older adults [14], fall status in healthy people [15], impact of chronic pain [16], or impaired postural control in patients with chronic obstructive pulmonary disease [17]. Although the clinical relevance of the kinematic performance in this test is well known, to our knowledge, there is a lack of studies that evaluate the test-retest reliability of these parameters, which may help to judge and interpret changes caused by an intervention or differences between populations. Therefore, studies are needed that report on the reliability, standard error of measurement, and smallest real difference (SRD) of kinematic data obtained during the execution of the 30 s Chair Stand test in different populations.

The main objective of the present study is to provide reliability parameters for the TUG and the 30 s Chair Stand test in healthy older adults. This study also aims to compare the results recorded using a manual stopwatch and an automatic chronometer in the TUG, as well as to report the reliability, standard error of measurement, and smallest real difference using each method.

## 2. Materials and Methods

### 2.1. Participants

A total of 99 healthy older adults participated in the study. The inclusion criteria were set as follows: (a) be aged 65 or more and (b) be able to walk autonomously without using any support. Participants were excluded when any of the following criteria was fulfilled: (a) be institutionalized or (b) suffer from a disease or injury that might affect the results in the tests. All participants understood and signed the written informed consent. The study was conducted in the elderly association from Montemor-o-Novo and the elderly center Airpiffs from Évora. The protocol of the study was approved by the Committee of Bioethics and Biosecurity of the University and is in agreement with the guidelines and values of the updated Helsinki Declaration and with the national legislation on bioethics, biomedical research, and personal data confidentiality (Code: GD/42998/2016; Date: 16/11/2016).

### 2.2. Procedure

**Figure 1:**
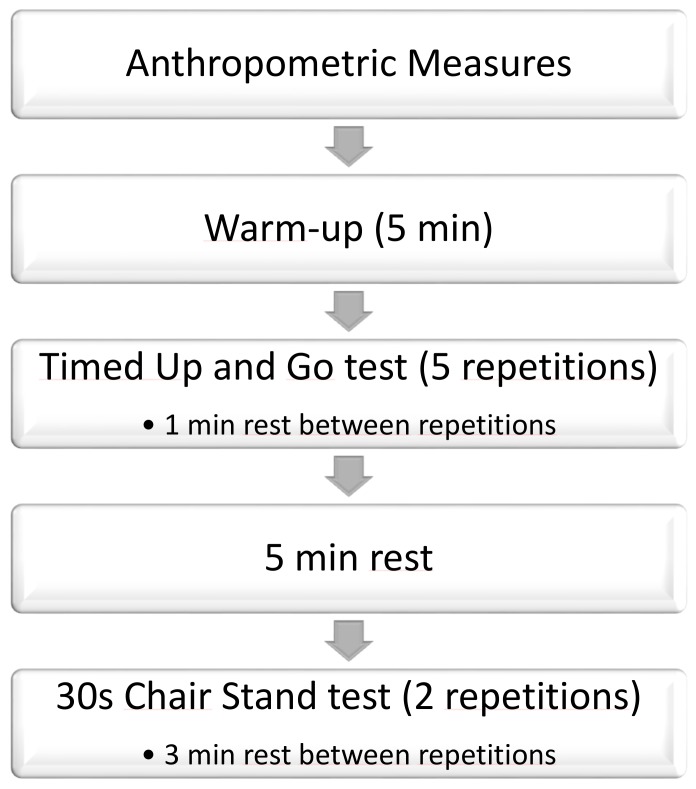
Summary diagram of the procedure.

First, anthropometrical measures were assessed using a SECA weighing device (SECA, Hamburg, Germany) and a measuring rod. Afterward, participants performed a light warm-up including self-paced walking and joint mobility for 5 min (see Figure 1).

All participants performed the TUG five times. The rest between each repetition and the subsequent one was one minute. Finally, five minutes after the end of the fifth repetition, participants were asked to perform the 30 s Chair Stand test. This test was carried out twice, with three minutes’ rest between each one.

In the TUG, an automatic chronometer was placed on the chair in order to assess the time required to complete the task. Specifically, the chronometer was the Chronopic (Chronojump, BoscoSystem^®^, Barcelona, Spain) [18]. This device is based on an electric circuit that can be opened and closed: when participants are touching the device wearing a vest with a metallic tape, the circuit is closed, whereas when participants lose contact with the device, the circuit is opened. The Chronopic tracks the amount of time the circuit is opened and closed. Therefore, in the TUG, participants started with their back touching the back support (circuit closed), then they stood up losing the contact (circuit open), and at the end of the task participants returned to the initial position (circuit closed again). A trained rater also measured the time manually with a stopwatch.

In the case of the 30 s Chair Stand test, the same device (Chronopic) was used to evaluate the time spent in each repetition. However, two phases were identified in the sit-to-stand-to-sit cycle: impulse phase, which is defined as the time elapsed from when the buttocks come into contact with the seat until the buttocks lose contact with the seat (i.e., all the time that the participant is seated) [14,16], and the no-contact phase, which is defined as the time elapsed from when the buttocks lose contact with the seat until the contact is made again. This phase comprises two phases defined by Millor et al. [14]: stand-up phase and sit-down phase.

### 2.3. Statistical Analysis

Descriptive statistics (mean and SD) of age and anthropometric measurements were calculated for the whole sample, men and women. Parametric and non-parametric tests were conducted based on the results of Kolmogorov–Smirnov tests. Differences between test and retest were evaluated using the paired samples *t*-test or Wilcoxon test when appropriate. Recommendations by Weir [19] were followed in order to evaluate the reliability of the tasks. The selected intraclass correlation coefficient (ICC) was 3,1 (two-way mixed, single measures). Absolute reliability was determined by computing the standard error of measurement, which is calculated as SEM = SD·$\sqrt{1-ICC}$, where SEM is the standard error of measurement and SD is the mean SD of the two repetitions (test and retest). The smallest real difference was also calculated as 1.96·SEM·$\sqrt{2}$. Both the standard error of measurement and the smallest real difference were converted into percentages to enable comparisons with further studies.

For the TUG, differences between the result obtained using a manual stopwatch and that recorded using an automatic chronometer were tested with repeated measures ANOVA and the Friedman test. Pairwise comparison analyses were performed through paired samples *t*-test and Wilcoxon test when appropriate. In these tests, pairwise comparisons between different repetitions were evaluated in order to provide reliability parameters according to the number of repetitions performed and to identify the optimum number of repetitions that should be performed. Therefore, statistical differences and reliability analyses were computed between each repetition and the consecutive one (1 vs. 2, 2 vs. 3, 3 vs. 4, and 4 vs. 5). Other comparisons (2 vs. 4, 2 vs. 5, and 3 vs. 5) were computed in order to draw conclusions about the most suitable number of repetitions. This procedure was performed with data from the manual stopwatch and also with results from the automatic chronometer. Moreover, Spearman’s Rho and Pearson’s r correlation coefficients were extracted to evaluate the relation between the results of the manual stopwatch and the automatic chronometer for each of the repetitions. 

All analyses were performed using SPSS v21 (IBM, Armonk, NY, USA) and Microsoft Excel 2013. Significance level was set at *p* < 0.05.

## 3. Results

Table 1 shows the main characteristics of the participants. Mean age (SD) was 70.63 (5.57) for men and 72.03 (6.83) for women. Regarding anthropometrical variables, women were shorter than men (162.03 vs. 172.55) and their weight was lower than that of men (70.97 vs. 80.38). There were no significant differences in body mass index (BMI) according to gender (26.98 vs. 27.05).

Reliability parameters obtained for the 30 s Chair Stand test are summarized in Table 2. Almost each variable achieved good reliability considering the classification by Munro et al. [20] (i.e., 0.70 to 0.90), except all parameters from the initial repetition and also the mean duration of the impulse phase from the last sit-to-stand-to-sit cycle of the test (<0.70). The smallest real difference oscillated between 13.1% for mean total duration of the last sit-to-stand-to-sit cycle and 50.50% for mean duration of the impulse phase from the first repetition of the test.

Table 3 shows the differences between the TUG assessed using an automatic chronometer and a manual stopwatch. Results point to the fact that there were some differences in repetitions but not in all of them.

Reliability parameters obtained for the Timed Up and Go test are summarized in Table 4. All the repetitions, for both automatic and manual chronometers, achieved good (>0.70) or excellent (>0.90) reliability considering the classification by Munro et al. [20]. The smallest real difference oscillated with the manual stopwatch between 12.21% in the comparison between the 2 and the 3 repetitions and 16.68% in the comparison between the 2 and the 5 repetitions for mean total duration of the Timed Up and Go test. In the automatic chronometer, the smallest real difference oscillated with the manual stopwatch between 11.50% in the comparison between the 2 and the 3 repetitions and 16.79% when comparing the 3 with the 5 repetitions. In addition, repeated measures ANOVA (F = 9.036; *p*-value < 0.001; η^2^ = 0.084) and the Friedman test (*p*-value < 0.001) showed a significant effect within the five repetitions with the manual stopwatch. In the same vein, the automatic chronometer, repeated measures ANOVA (F = 5.885; *p*-value <0.001; η² = 0.057) and the Friedman test (*p*-value < 0.001), showed a significant effect within the five repetitions. Pairwise analyses are detailed in Table 4. Significant between-repetition differences only occurred when the first and the fifth repetitions were included. There were no differences among repetitions 2, 3, and 4. 

## 4. Discussion

The main finding of the present study is that the 30 s Chair Stand test and the TUG are reliable in healthy older adults. Furthermore, this study provides the smallest real difference not only for the crude results of the tests, but also for the sit-to-stand-to-sit cycle, impulse phase and no-contact phase at the beginning and at the end of the 30 s task. Results indicate that the number of repetitions and mean durations of the total sit-to-stand-to-sit cycle, impulse phase and no-contact phase had very good reliability (ICC between 0.80 and 0.90). However, the reliability of those variables when analyzing the beginning or the end of the task was not always good and should be used with caution.

As expected, reliability was always better when the automatic chronometer was used to evaluate the TUG. These results are in line with those reported by Collado-Mateo et al. [12], who stated that using an automatic chronometer to assess the TUG in women with fibromyalgia increased the ICC and reduced the standard error of measurement and the smallest real difference. The relevance of the TUG in the elderly is well known, being one of the physical fitness tests which better correlates with health-related quality of life [21].

The use of an automatic chronometer may be a low-cost alternative to assess kinematic parameters in physical fitness tests, providing additional data that may be relevant for clinicians and researchers. The present study showed that it is possible to assess the impulse phase using this kind of device, achieving good reliability (ICC = 0.821). Lack of muscle strength could cause an increment in the duration of the impulse phase during the whole 30 s task. This could be an issue among older adults since the difference between the first and the last repetitions was large, that is, the impulse phase changed from 1.09 s in the first cycle and 1.33 s in the last (22% increase), while the no-contact phase increased from 1.94 s to 2.02 s (4% increase). Therefore, further studies may investigate the relevance of the kinematic performance over the 30 s Chair Stand test. In this regard, fatigability is increased in older adults and may lead to a high risk of mobility loss and a risk of falling [22]. Lindemann et al. [23] also pointed out the relevance of this evaluation and suggested a protocol based on the velocity of the sit-to-stand performance. That protocol may be complementary to a protocol based on duration of phases or cycles since the automatic chronometer and the linear encoder may be used simultaneously.

Another relevant contribution of the present study is the calculation of reliability for five repetitions of the TUG. Most of the studies in the literature chose performing two or three repetitions and recording the second, the third, the mean, or the best repetitions. Our results clearly indicate that the first repetition may be considered as familiarization, since the mean time is significantly higher than the second repetition. Similarly, the fifth repetition is significantly higher than the previous one. This could be due to the resting time, which was 1 min in this study. Thus, fatigue may appear after four repetitions with a 60 s rest between them in older adults. Therefore, according to the results from Table 3 and Table 4, a minimum of two and a maximum of four repetitions should be performed in this population. There was no significant change from the second to the third repetition, nor from the third to the fourth. Furthermore, the third repetition was the best of the five repetitions. In summary, these results may indicate that the third repetition is the most adequate to be used as the final score in the TUG. Another adequate alternative would be to compute the average of the second, the third and the fourth repetitions.

Some limitations may be mentioned in this study. First, the sit-to-stand-to-sit cycle could only be divided into two phases (impulse phase and no-contact phases) using the Chronopic device. Thus, other relevant phases such as sit-to-stand or stand-to-sit could not be identified since the key point for this division is the standing position. Second, the height of the chair was the same for all participants regardless of their gender or height. Most studies using these tests follow this protocol, but it is known that the height of the chair might alter the kinematic performance of the sit-to-stand-to-sit cycle [24].

## 5. Conclusions

The reliability of the TUG and the 30 s Chair Stand test is good in healthy older adults. Using an automatic chronometer is possible to identify relevant and reliable phases in the sit-to-stand-to-sit cycles from the 30 s Chair Stand test. Furthermore, the reliability of the TUG can be improved by increasing the ICC and reducing the smallest real difference and the standard error of measurement when the result is assessed using that device. Therefore, the use of an automatic chronometer is recommended to evaluate the 30 s Chair Stand test and the TUG in the elderly.

## Figures and Tables

**Table 1:** Main characteristics of the participants.

	All (*n* = 99)	Men (*n* = 66)	Women (*n* = 33)
Age (years)	71.10 ± 6.02	70.63 ± 5.57	72.03 ± 6.83
Height (cm)	169.04 ± 8.66	172.55 ± 6.85	162.03 ± 7.66
BMI (kg/cm^2^)	27.00 ± 2.51	26.98 ± 2.54	27.05 ± 2.48
Weight (kg)	77.24 ± 10.09	80.38 ± 9.23	70.97 ± 8.83

**Table 2:** Test-retest reliability of the 30 s Chair Stand test (*n* = 99).

	Mean ± SD Test	Mean ± SD Retest	*p*-Value	Distribution Value	ICC (95% CI)	SEM	SEM (%)	SRD	SRD (%)
Number of repetitions	9.99 ± 1.84	9.78 ± 1.96	0.047	<0.001	0.874 (0.817–0.913)	0.67	6.82	1.87	18.91
Mean duration of sit-to-stand-to-sit cycle (s)	3.21 ± 0.63	3.28 ± 0.70	0.164	<0.001	0.889 (0.838–0.924)	0.67	20.5	1.84	56.8
Mean duration of initial sit-to-stand-to-sit cycle (s)	3.03 ± 0.65	3.13 ± 0.64	0.015	<0.001	0.675 (0.551–0.769)	0.21	7.0	0.60	19.3
Mean duration of last sit-to-stand-to-sit cycle (s)	3.36 ± 0.84	3.41 ± 0.83	0.411	0.135	0.771 (0.676–0.840)	0.48	4.7	1.32	13.1
Mean duration of impulse phase (s)	1.22 ± 0.22	1.27 ± 0.26	<0.001	0.892	0.821 (0.745–0.876)	0.10	8.16	0.28	22.61
Mean duration of the initial impulse phase (s)	1.09 ± 0.25	1.16 ± 0.26	0.003	0.036	0.354 (0.169–0.515)	0.20	18.22	0.57	50.50
Mean duration of the last impulse phase (s)	1.33 ± 0.32	1.38 ± 0.30	0.019	0.010	0.676 (0.553–0.771)	0.18	13.02	0.49	36.10
Mean duration of the no-contact phase(s)	1.99 ± 0.49	2.00 ± 0.55	0.822	0.005	0.884 (0.833–0.921)	0.18	8.88	0.49	24.61
Mean duration of the initial no-contact phase (s)	1.94 ± 0.46	1.97 ± 0.51	0.752	<0.001	0.699 (0.582–0.787)	0.27	13.61	0.74	37.73
Mean duration of the last no-contact phase	2.02 ± 0.67	2.03 ± 0.69	0.985	0.008	0.715 (0.603–0.799)	0.36	17.93	1.01	49.69

ICC, intraclass correlation coefficient; SEM, standard error of measurement; SRD, smallest real difference. Paired *t*-tests or Wilcoxon tests were conducted depending on the distribution (variables with a *p*-value lower than 0.05 in the Shapiro–Wilk test were considered for a non-parametric analysis).

**Table 3:** Mean and SD of the five repetitions of the Timed Up and Go test (TUG) (*n* = 99).

	Repetition 1	Repetition 2	Repetition 3	Repetition 4	Repetition 5
TUG manual stopwatch (s)	9.92 ± 1.69	9.67 ± 1.59	9.57 ± 1.59	9.62 ± 1.67	9.78 ± 1.72
TUG automatic chronometer (s)	10.09 ± 1.68	9.65 ± 1.64	9.62 ± 1.65	9.69 ± 1.73	9.72 ± 1.79
Distribution *p*-value	0.198	0.015	0.120	0.722	0.113
Paired sample comparisons *p*-value	<0.001 *	0.662	0.085	0.020	0.122
Correlation coefficient	0.979 *	0.966 *	0.987 *	0.984 *	0.982 *

* *p*-value lower than 0.001. TUG, Timed Up and Go test. Paired *t*-tests or Wilcoxon tests were conducted depending on the distribution (variables with a *p*-value lower than 0.05 in the Shapiro–Wilk test were considered for a non-parametric analysis). For correlation analyses, Spearman’s Rho or Pearson’s r were used when appropriate.

**Table 4:** Test-retest reliability of the Timed Up and Go test (*n* = 99).

Repetitions	Distribution *p*-Value	*p*-Value	ICC (95% CI)	SEM (Nm)	SEM (%)	SRD (Nm)	SRD (%)
	**Manual Stopwatch**						
1 vs. 2	0.440	<0.001	0.878 (0.825–0.916)	0.57	5.85	1.59	16.21
2 vs. 3	0.231	0.619	0.929 (0.896–0.952)	0.42	4.40	1.17	12.21
2 vs. 4	0.003	0.893	0.879 (0.825–0.917)	0.57	5.88	1.57	16.29
2 vs. 5	0.016	0.242	0.875 (0.819–0.914)	0.59	6.02	1.62	16.68
3 vs. 4	0.154	0.353	0.894 (0.846–0.928)	0.53	5.53	1.47	15.33
3 vs. 5	0.014	0.227	0.866 (0.807–0.908)	0.51	5.22	1.40	14.47
4 vs. 5	0.018	0.787	0.892 (0.843–0.926)	0.47	4.81	1.29	13.34
	**Automatic Chronometer**						
1 vs. 2	0.249	0.001	0.892 (0.843–0.926)	0.55	5.53	1.51	15.32
2 vs. 3	0.139	0.075	0.941 (0.913–0.960)	0.40	4.15	1.11	11.50
2 vs. 4	0.008	0.392	0.908 (0.866–0.937)	0.51	5.29	1.42	14.65
2 vs. 5	<0.001	0.122	0.896 (0.848–0.929)	0.55	5.71	1.53	15.83
3 vs. 4	0.033	0.999	0.908 (0.867–0.938)	0.51	5.31	1.42	14.72
3 vs. 5	0.002	0.016	0.884 (0.833–0.921)	0.59	6.06	1.62	16.79
4 vs. 5	0.001	0.038	0.894 (0.846–0.927)	0.57	5.90	1.59	16.37

ICC, intraclass correlation coefficient; SEM, standard error of measurement; SRD, smallest real difference. Paired *t*-tests or Wilcoxon tests were conducted depending on the distribution (variables with a *p*-value lower than 0.05 in the Shapiro–Wilk test were considered for a non-parametric analysis).
